# Whole genome and transcriptome sequencing of matched primary and peritoneal metastatic gastric carcinoma

**DOI:** 10.1038/srep13750

**Published:** 2015-09-02

**Authors:** J. Zhang, J. Y. Huang, Y. N. Chen, F. Yuan, H. Zhang, F. H. Yan, M. J. Wang, G. Wang, M. Su, G Lu, Y. Huang, H. Dai, J. Ji, J. Zhang, J. N. Zhang, Y. N. Jiang, S. J. Chen, Z. G. Zhu, Y. Y. Yu

**Affiliations:** 1 Shanghai Institute of Digestive Surgery and Biobank of Gastrointestinal carcinoma, Lu Wan Qu, Shanghai, China; 2 Collaborative Innovation Center of Systems Biomedicine, China; 3 State Key Laboratory of Medical Genomics, Changsha City, Hunan Prov, China; 4 Department of Pathology of Shanghai Ruijin Hospital, Shanghai Jiao Tong University School of Medicine, 200025, Shanghai, China; 5 Department of Radiology of Shanghai Ruijin Hospital, Shanghai Jiao Tong University School of Medicine, 200025, Shanghai, China; 6 Genome Center of WuXiAppTec (Shanghai) Co., Ltd. Shanghai, 200131, China

## Abstract

Gastric cancer is one of the most aggressive cancers and is the second leading cause of cancer death worldwide. Approximately 40% of global gastric cancer cases occur in China, with peritoneal metastasis being the prevalent form of recurrence and metastasis in advanced disease. Currently, there are limited clinical approaches for predicting and treatment of peritoneal metastasis, resulting in a 6-month average survival time. By comprehensive genome analysis will uncover the pathogenesis of peritoneal metastasis. Here we describe a comprehensive whole-genome and transcriptome sequencing analysis of one advanced gastric cancer case, including non-cancerous mucosa, primary cancer and matched peritoneal metastatic cancer. The peripheral blood is used as normal control. We identified 27 mutated genes, of which 19 genes are reported in COSMIC database (*ZNF208, CRNN, ATXN3, DCTN1, RP1L1, PRB4, PRB1, MUC4, HS6ST3, MUC17, JAM2, ITGAD, IREB2, IQUB, CORO1B, CCDC121, AKAP2, ACAN* and *ACADL*), and eight genes have not previously been described in gastric cancer (*CCDC178, ARMC4, TUBB6, PLIN4, PKLR, PDZD2, DMBT1*and *DAB1*).Additionally,*GPX4* and *MPND* in 19q13.3-13.4 region, is characterized as a novel fusion-gene. This study disclosed novel biological markers and tumorigenic pathways that would predict gastric cancer occurring peritoneal metastasis.

Gastric cancer is the second leading cause of cancer-related death worldwide. Many patients with advanced gastric cancer suffer from tumor recurrence and metastasis following curative gastrectomy, even if no residual tumor remains after surgical resection. Peritoneal metastasis (PM) represents one of the most common causes of failure after curative surgery for gastric cancer, constituting 20%–53.5% of recurrences^1^,^2^,^3^. Therefore, it is important to identify patients at a high risk of PM. However, it is difficult to predict PM using common clinicopathological factors. It is desirable to find new predictive factors of PM in advanced gastric cancer. As we know, the prognosis for gastric cancer with PM is the worst among its recurrence patterns, with a mean survival of less than 6 months^4^. However, the molecular pathogenesis by which gastric cancer undergoes PM remains mostly unknown. Given the detrimental influence of PM on survival, efforts should be undertaken to explore the possible molecular mechanisms and preventing or treating strategies.

Recently, next generation sequencing (NGS) has become very useful tools of comprehensive research on cancer, facilitating the identification of treatment targets and personalized treatments. NGS can systematically identify gene alterations for cancer and is a powerful approach for investigating the carcinogenesis and identifying novel therapeutic targets, as well as biomarkers^5^,^6^,^7^. Over the past few years, advances of NGS and affordable price have resulted in increased cancer genome studies, which are of great helpfulness to investigate pathogenesis, driver genes, molecular classification and drug targets for human gastric cancer^8^. For instance, Wang and Zang’s groups separately revealed a number of potential cancer-driving genes of gastric cancer by whole-exome sequencing and identified recurrent somatic mutations in the chromatin remodeling gene *ARID1A* and alterations in the cell adhesion gene *FAT4*^9^. Wang′s group also performed whole-genome sequencing in 100 tumor-normal pairs of gastric cancer and identified significantly mutated driver genes (*MUC6, CTNNA2, GLI3, RNF43* and others)^10^. Cancer Genome Atlas Research Network published their genome research for 295 primary gastric adenocarcinomas and proposed a molecular classification dividing gastric cancer into four subtypes. That is tumors positive for Epstein-Barr virus, microsatellite unstable tumors, genomically stable tumors and chromosomal instability tumors. Identification of these subtypes provides a roadmap for patient stratification and trials of targeted therapies^11^. Nagarajan and colleagues analyzed whole-genome of two gastric adenocarcinomas, one with chromosomal instability and the other with microsatellite instability and revealed microsatellite instability-related mutational signatures^12^. Liu and colleagues discovered the spectrum of genomic and transcriptomic alterations in gastric cancer and *ZAK* kinase isoform^13^.

On cancer genome research, the high-quality samples are the critical factor. Since gastric cancer with PM was considered as an incurable stage at surgery before, so, it was nearly impossible to get primary tumor and paired PM tumor samples at the same time^14^. Recently, non-curative resection of PM has been introduced into treatment of gastric cancer with single peritoneal metastasis. Xia and colleagues proposed that the overall survival of patients in the non-curative resection group was longer (14.869 months) than that in the non-resection group (7.780 months). Non-curative resection of PM significantly prolonged survival of gastric cancer patients^15^. Along with the change of therapeutic mode, it becomes possible to get primary tumor sample and paired PM sample simultaneously. Here, we describe the clinicopathological features and whole-genome and transcriptome sequencing characterization on a set of precious samples from a paired gastritis, primary gastric cancer, peritoneal metastasis, as well as peripheral blood. Genome-wide screening of whole genome and transcriptome dysregulation between non-cancerous tissues, primary cancer and PM tumor would provide insights into the molecular basis of gastric cancer initiation, progression and metastasis.

## Results

### Clinicopathology of the case

**Figure 1:**
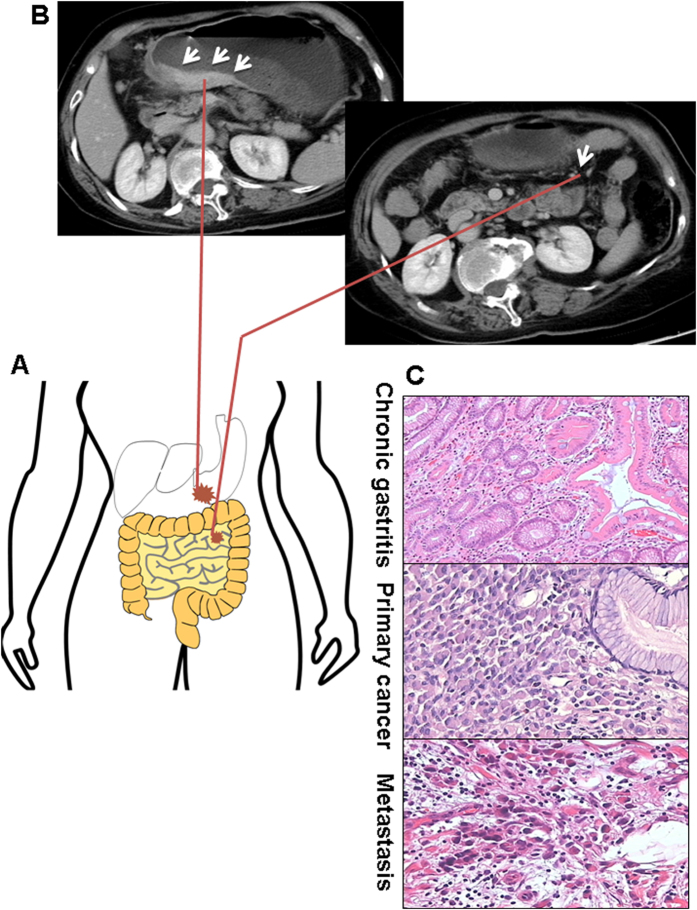
Clinical information of the patient. (**A**) Schematic diagram of gastric cancer with peritoneal metastasis on mesocolon transversum drawn by Yu YY. (**B**) Left, CT scan shows that the tumor located on antrum with penetration of serosa (white arrow). The stomach is filled with liquid by pyloric obstruction. Right, the metastatic tumor is noticed on the surface of mesocolon transversum (white arrow). (**C**) Microscopic features of chronic gastritis (top), primary gastric cancer (middle) and metastatic tumor on mesocolon transversum (down) by hematoxilin and eosin (H&E) stain of the case.

The subject was diagnosed as gastric cancer occurring peritoneal metastasis before operation by computerized tomography (CT) scan of abdominal region as well as intraoperative observation (Fig. 1A,B). No other organs metastases except peritoneum were found before operation based on systematic examination. No neoadjuvant or adjuvant chemotherapy was administered before operation. Macroscopically, the tumor located in antrum with diameter 7 centimeters, Borrmann III type. The histological examination after total gastrectomy and noncurative resection of metastatic tumor demonstrated the tumor extensively infiltrates in gastric wall with penetration of serosa. Tumor cells revealed signet ring cell histology (Fig. 1C, middle). The PM tumor also disclosed signet ring cell histology (Fig. 1C, down). The non-cancerous mucosa revealed chronic gastritis histology (Fig. 1C, top). The final diagnosis was poor-differentiated gastric cancer, diffuse type, with PM. There was significant metastasis in perigastric lymph nodes (18/28). The patient was classified as stage IV, a late stage of gastric cancer. The patient died one month after operation.

### Whole genome sequencing (WGS)

Sequencing of the matched peripheral blood, gastritis, primary cancer and PM cancer was performed. The sequencing yielded average 167.75 Gb for above samples. The reads were mapped to the reference sequences of human genome hg19 and covered about 99.08% of the reference genome with mean 57× (range 34–80×)sequencing depth. The details of WGS were listed in Supplementary table 1.

**Figure 2:**
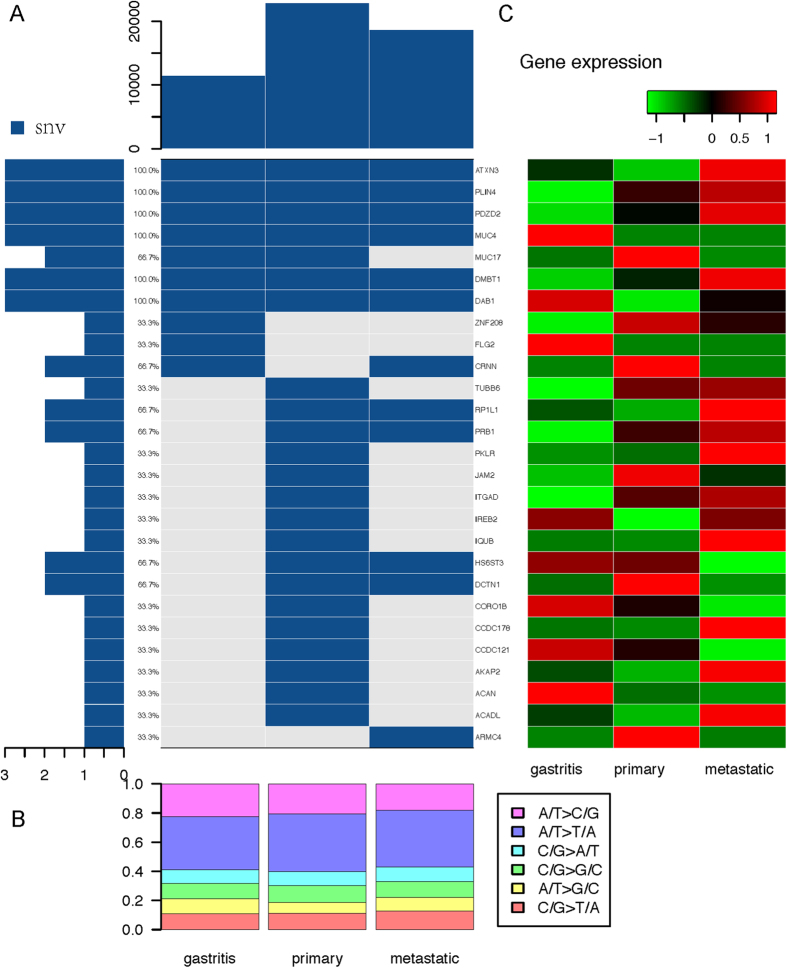
Correlation of somatic mutations and gene expression of chronic gastritis, primary cancer and metastatic cancer tissues. (**A**) Top panel shows a summary of all kinds of somatic mutations in chronic gastritis, primary cancer and metastatic cancer tissues. The matrix in the center of the figure represents individual mutations in each tissue. Blue represents single nucleotide variation. Left bar chart indicates number of mutations for each gene. Percentages represent the fraction of tumors harboring at least one mutation in the specified gene. (**B**) The bars show percentage of somatic SNVs identified by whole genome sequencing in chronic gastritis, primary cancer and metastatic cancer tissues, compared to peripheral blood. (**C**) Heatmap plot shows gene expression levels from RNA-seq for mutated genes in chronic gastritis, primary cancer and metastatic cancer tissues.

Taking the blood sample as normal reference, we identified somatic alterations throughout the genome, all of which are single nucleotide variants (SNVs). The somatic alterations were not only found in primary cancer and PM cancer, but also in chronic gastritis mucosa far from primary tumor (Supplementary table 2). A total of 27 somatic variations associated to amino acid alteration were found (All somatic variations are validated by conventional Sanger sequencing). From which, 10 somatic variation were detected on chronic gastritis (*ATXN3, PLIN4, PDZD2, MUC4, MUC17, DMBT1, DAB1, ZNF208, FLG2* and *CRNN*), 23 somatic variations were occurred in primary cancer (*ATXN3, PLIN4, PDZD2, MUC4, MUC17, DMBT1, DAB1, TUBB6, RP1L1, PRB1, PKLR, JAM2, ITGAD, IREB2, IQUB, HS6ST3, DCTN1, CORO1B, CCDC178, CCDC121, AKAP2, ACAN* and *ACADL*), and 12 somatic variations in metastatic cancer (*ATXN3, PLIN4, PDZD2, MUC4, DMBT1, DAB1, CRNN, RP1L1, PRB1, HS6ST3, DCTN1* and *ARMC4*). We noticed that 6 somatic variations (*ATXN3, PLIN4, PDZD2, MUC4, DMBT1* and *DAB1*) were simultaneously observed in triple samples of chronic gastritis, primary cancer and PM cancer. Four somatic variations (*RP1L1, PRB1, HS6ST3* and *DCTN1*) were simultaneously occurred in both primary tumor and PM cancer. One somatic variation (*ARMC4*) was only observed in PM cancer (Fig. 2A). In terms of nucleotide substitution, the average proportion of transitions in gastritis, primary and metastatic cancer was 58.8%, 60.1% and 57.0%. The most prevalent changes were A → G/T → C transitions, followed by G → A/C → T transitions and A → C/T → G transitions (Fig. 2B). By comparing the transitions with TCGA dataset, the results show the prevalent changes are similar to other cancer types.

### Effect of nonsynonymous coding SNVs on gene expression

As shown in heatmap plot of Fig. 2C, some mutated genes resulted in down-regulation of gene expression, and some resulted in up-regulation of gene expression. In order to explore the possible predicting biomarkers of PM, we specially concerned with the mutations simultaneously occurred in both primary cancer and PM cancer. Among them, the somatic mutation of *RP1L1* and *PRB1* caused gene activation with elevated mRNA expression in primary cancer, PM cancer or in both. They are noticeable molecular targets for tumor metastasis. In addition, gene *TUBB6* is mutated in primary cancer, accompanied by highly expressed *TUBB6* mRNA in both primary cancer and metastatic cancer relatively to gastritis tissue (Fold change > 6). Regard to PM specific nonsynonymous mutation, *ARMC4* mutation resulted in the decreased level of mRNA expression.

### Analysis of fusion transcripts

**Figure 3:**
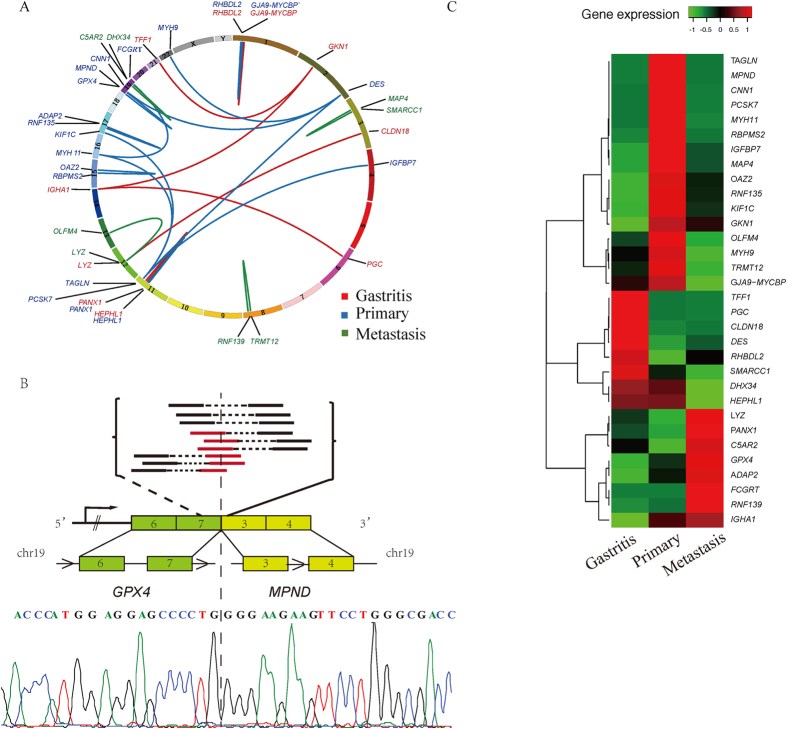
*GPX4-MPND* gene fusion. (**A**) Circos plot of multiple fusion genes identidied in chronic gastritis (red inner lines), primary cancer (blue inner lines) and PM cancer (green inner lines). The arabic numbers represent chromesome numbers. The nemes of fusion genes labled outside the circs. (**B**) Schematic diagram of fusion site for *GPX4* and *MPND* genes. Schematic of the predicted gene fusion illustrating RNA-seq evidence that support the fusion between exon 7 of *GPX4* and exon 3 of *MPND*. Reads highlighted in red span across the fusion junction. The Sanger sequecing result of fusion junction is also provided. (**C**) Heatmap plot shows gene expression levels from RNA-seq for fusion genes identified in chronic gastritis, primary cancer and metastatic cancer tissues.

Base on the fusion detecting module of TopHat, we searched the gene fusion in each sample. Several potential gene fusions were found and the circos plot was used to present the existence and locations of gene fusion along the chromosomes (Fig. 3). We excluded the fusions that the distances of two genes were less than 100 kb, since these kinds of fusions may be artificial. We listed fusion genes of cancer samples and their altered mRNA expression levels compared to gastritis in Table 1. All the fusion genes shown in Fig. 3A were verified by Sanger sequencing, but positive validation result was got in *GPX4-MPND* fusion gene pair[*GPX4*(chr19:1106458)-*MPND*(chr19:4345742)]. By RNA-seq analysis, this fusion event resulted in elevated mRNA expression in both genes. The mRNA expression of *GPX4* gene is increased 2.184-fold (primary cancer vs gastritis), and mRNA expression of *MPND* increased 1.3369-fold (primary cancer vs gastritis).

### Differential expressed genes and pathways

**Figure 4:**
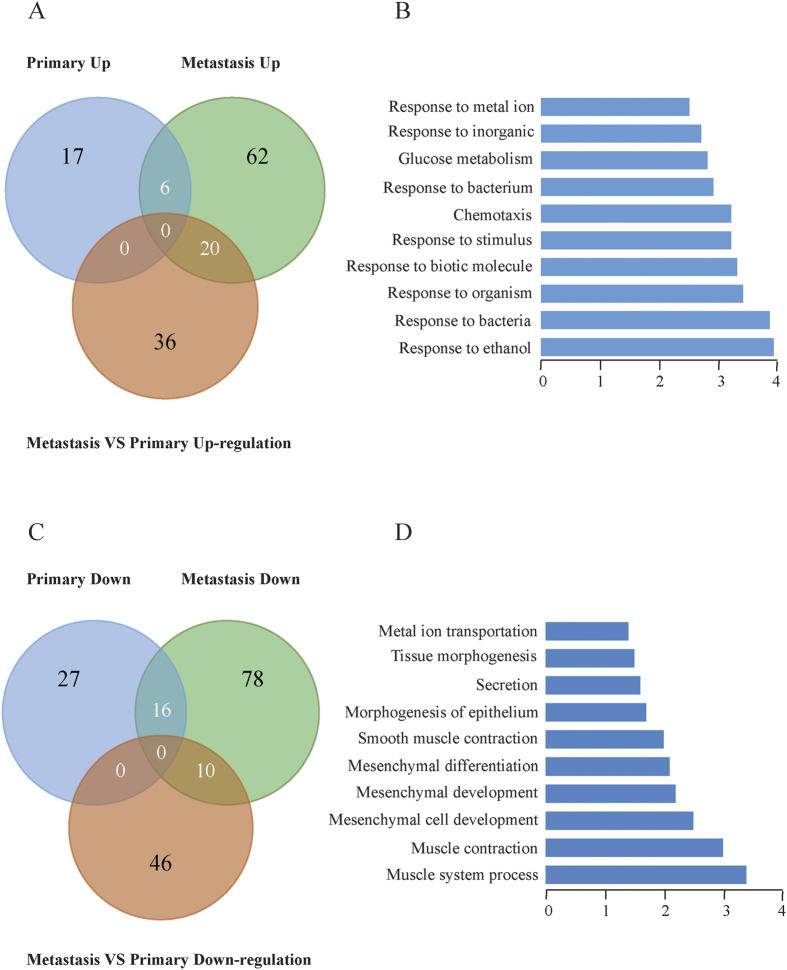
Differential expressed genes and involved pathways in chronic gastritis, primary cancer and PM cancer. (**A**) The overview of up-regulated genes of primary cancer or PM cancer relative to chronic gastritis. (**B**) Pathway analysis of shared up-regulated genes in both primary cancer and PM cancer by GO enrichment. (**C**) The overview of down-regulated genes of primary cancer or PM cancer relative to chronic gastritis. (**D**) Pathway analysis of shared down-regulated genes in both primary cancer and PM cancer by GO enrichment.

We analyzed differentially expressed genes upon FDR < 0.1 and fold-change >1.5. We compared the differences of primary cancer vs chronic gastritis, metastatic cancer vs chronic gastritis, metastatic cancer vs primary cancer, separately. The overview of the accounts of differential expressed genes for each sample was shown in Fig. 4. To find the crucial gene cluster that plays a key role in PM of gastric cancer, we paid attention to the shared genes both in primary cancer and metastatic cancer and listed them in Table 2. We noticed 6 genes (*SFRP4, NOX4, HOXA11, NKX2-5, CDH16* and *LOC100505875*) were special up-regulated both in primary cancer and PM cancer, and 16 genes (*LIPF, NKX6-2, MIXL1, CWH43, SULT1E1, CXCL5, REG1A, GHRL, NKX2-2, HTR1E, HPGD, ESRRG, CYP2C19, ADH1C, PNLIPRP3* and *CEACAM5*) were special down-regulated both in primary cancer and PM cancer. Using Gene Ontology (GO) functional annotation, signaling pathways was analyzed on the shared genes occurred in both primary cancer and PM. Compared to chronic gastritis, there was significant down-regulation of epithelium morphogenesis, secretion and muscle development-related genes both in primary cancer and PM cancer. Whereas, compared to chronic gastritis, there was significant up-regulation of genes related to response to bacteria, response to ethanol, response to stimulus, chemotaxis and glucose metabolism both in primary cancer and PM cancer.

## Discussion

Here we report comprehensive characteristics of whole genome and transcriptome of a typical case of diffuse type gastric carcinoma with PM. Diffuse type gastric carcinoma accounts for about 40% of all gastric cancers, which is characterized by extensive poorly cohesive cells infiltration in stomach or metastatic sites. Diffuse type gastric carcinoma frequently develops into PM, consisting of peritoneal dissemination of cancer cells, and leads to extremely poor prognosis^16^,^17^. Although several molecules have been reported to be involved in the PM of gastric cancer, the mechanisms underlying poorer biological behavior have yet to be elucidated. Thus, systematic analysis of genomic and transcriptomic variant profiling of PM is essential. Recently, we collected a precious case, which covers matched peripheral blood, non-cancerous mucosa, primary cancer and PM cancer. WGS and RNA-seq were used to screen high-risk genes variations for occurring PM in gastric cancer. To our knowledge, this is first genomic sequencing study simultaneously for primary cancer and PM cancer worldwide.

At WGS level, we found a set of nonsynonymous mutation genes occurred in chronic gastritis sample, they are *ATXN3, PLIN4, PDZD2, MUC4, MUC17, DMBT1, DAB1, ZNF208, FLG2* and *CRNN.* It means that somatic gene variations are an accumulative molecular event during early stage of gastric carcinogenesis. In order to find out predictive molecular markers for PM of gastric cancer, we pay attention to the mutation genes simultaneously occurred in both primary cancer and PM cancer. For instance, the somatic variations of *RP1L1, PRB1, HS6ST3* and *DCTN1* were simultaneously observed in both primary tumor and PM cancer. They are very noticeable molecular targets for understanding peritoneal metastasis on gastric cancer. *RPIL1* is a retinitis pigmentosa 1-like1 gene, and no cancer-associated report yet. *PRB1* encodes proline-rich protein BstNI subfamily 1. There is no cancer-associated report yet. *HS6ST3* encodes heparan sulfate 6-O-sulfotransferase 3 and is implicated in proliferation, differentiation, adhesion, migration, inflammation, blood coagulation, and other diverse processes. *HS6ST3* was found highly expressed in chondrosarcomas^18^. *DCTN1* encodes the dynactin, which binds to both microtubules and cytoplasmic dynein and is involved in diverse cellular functions. There is no cancer-associated report for *DCTN1* yet. *ARMC4* is firstly identified PM-specific somatic variation. There is also no cancer-associated report for *ARMC4*. In current case, the frequency of somatic variation in primary cancer is higher than that in metastatic cancer. This may reflect the higher heterozygosity of tumor cells in primary tumor, while the metastatic fraction may come from a subclone of primary tumor cells.

RNA-seq is employed for measuring global gene expression in order to determine the impact of genetic variants on gene functions. We integrated the WGS with RNA-seq together and found that some nonsynonymous mutations could cause elevation of gene activity with up-regulation of mRNA expression (*RP1L1*, *PRB1* and *TUBB6*). Although there is no cancer-related report for *RP1L1* and *PRB1* yet, *TUBB6* was found as a candidate oncogene in colorectal carcinoma^19^,^20^. Some nonsynonymous mutations caused low levels of gene activity with down-regulation of mRNA expression (*ZNF208, CRNN, IREB2* and *ACADL*), which may be crucial tumor suppressor genes for gastric cancer and may contribute to the extensive dissemination of cancer cells in abdominal cavity.

Furthermore, we filtered the intra-chromosome short distance (less than 100 kb) fusion and found out a set of fusion genes. Based on RNA-seq, we noticed some fusion genes accompanied alteration of mRNA expression levels. However, by Sanger sequencing validation, the positive validation result of fusion gene was got only in *GPX4-MPND* fusion gene pair. The gene fusion of *GPX4-MPND* causes up-regulations of mRNA expression of both genes in primary cancer. Up-to-data, there is no any report on *GPX4-MPND* gene fusion in cancer. *GPX4*(ID:2879, 19p13.3) gene encodes a member of the glutathione peroxidase family (22.5KD) and catalyzes the reduction of hydrogen peroxide, organic hydroperoxide, and lipid peroxides by reduced glutathione. *GPX4* plays a role on cell protection against oxidative damage. The association of *GPX4* with the human cancer is controversial. Some authors proposed that *GPX4* is decreased in cancer tissues, but others found a diverse function of *GPX4* on cancer^21^,^22^,^23^. *MPND* gene (ID: 84954, 19p13.3) was cloned from retinoblastoma and encodes a 48.5 KD protein of deubiquitinase family. Up-to-date, it is short of report for its correlation with carcinogenesis. We speculate that increased levels of *GPX4* and *MPND* in primary cancer may facilitate tumor growth and progression. Actually, we presented the fact that the changes of mRNA expression could occur in both genes of the fusion gene pair, but not only in the down-stream gene of the fused pair.

Upon RNA-seq, we identified a set of differentially expressed genes in different sites. We especially noticed the shared genes between primary cancer and metastatic cancer. We considered they are noticeable molecular targets for gastric cancer with PM. Among 22 shared genes, *SFRP4, NOX4, HOXA11, NKX2-5, CDH16* and *LOC100505875* were up-regulated in primary cancer and PM cancer, and *LIPF, NKX6-2, MIXL1, CWH43, SULT1E1, CXCL5, REG1A, GHRL, NKX2-2, HTR1E, HPGD, ESRRG, CYP2C19, ADH1C, PNLIPRP3* and *CEACAM5* were down-regulated in both sites. *SFRP4* encodes a secreted frizzled-related protein 4, and act as soluble modulators of Wnt signaling. Researchers found that *SFRP4* was overexpressed in colorectal cancer^24^. *NOX4* encodes a member of the *NOX*-family of enzymes that functions as the catalytic subunit the NADPH oxidase complex. NOX4 protein is localized to non-phagocytic cells where it acts as an oxygen sensor and catalyzes the reduction of molecular oxygen to reactive oxygen species. Hiraga and coworkers reported that *NOX4* was up-regulated in pancreatic cancer, and contributed to *TGF*-beta-induced epithelial-mesenchymal transition^25^. Zhang and coworkers found that *NOX4* was up-regulated in non small cell lung cancer, and promoted its growth and metastasis^26^. *HOXA11* encodes a homeobox transcription factor, which may regulate gene expression, morphogenesis, and differentiation. Cui and colleagues found that there were epigenetic changes of *HOXA11* in gastric cancer^27^. *NKX2-5* encodes a homeobox transcription factor and functions in heart formation and development. Moussa and Sidhom proposed that *NKX2-5* was partially up-regulated in T-cell acute lymphoblastic leukemia^28^. *CDH16* encodes a calcium-dependent membrane-associated glycoprotein and functions as the principal mediator of homotypic cellular recognition. It plays a role in morphogenic direction of tissue development. Di Martino and coworkers found that *CDH16* is one of the downstream targets of *FGFR3*, and involved in regulating cell-cell and cell-matrix adhesion in urothelial cells^29^.

In conclusion, this is the first report that WGS and RNA-seq are used for a set of matched samples from a gastric cancer case. The shared somatic mutations of primary cancer and metastatic cancer, as well as PM-specific somatic mutation are found out. Some mutations result in an activation of genes accompanied by up-regulation of gene expression; whereas, others result in an inactivation of genes, accompanied by down-regulation of gene expression. The former may be promise predictive markers for peritoneal metastasis of gastric cancer, while the latter may be important metastasis suppressor gene in gastric cancer. *GPX4-MPND* fusion gene is a novel molecular event identified in gastric cancer. This gene fusion may activate the gene pair and facilitates cancer growth and invasion.

## Methods

### Samples

The tissue samples were collected from a 70-year-old female. Computerized tomography scan of the abdominal region revealed that tumor was located at antrum resulting in pyloric obstruction. The tumor revealed obvious penetration of gastric wall and extended to perigastric tissues. There were metastatic nodules on mesocolon transversum. The patient underwent total gastrectomy and noncurative resection of metastatic tumor of mesocolon transversum. The tissue sample from non-cancerous mucosa was at least 5 cm from primary cancer. Primary cancer and peritoneal metastatic nodule were resected immediately frozen in liquid nitrogen and stored at −80 °C until DNA and RNA extraction. Independent pathological review of primary cancer and peritoneal metastasis of the samples with hematoxylin and eosin (H&E) staining was proceeded by two pathologists (F.Y and YY.Y) and confirmed the diagnosis of gastric cancer, diffuse-type with signet-ring cells, both for primary cancer and peritoneal metastatic cancer. Both samples from primary cancer and peritoneal metastatic cancer disclosed 70% pure of tumor cells. Written informed consent was obtained from the patient. The study was approved by the institutional review board of Ruijin Hospital, Shanghai Jiao Tong University School of Medicine and all the methods were carried out in accordance with the approved guidelines.

### Whole genome sequencing

Genomic DNA was extracted from the tissue specimens using QIAamp DNA kit (Qiagen, Germany). Genome DNA sequencing libraries were constructed using TruSeq DNA LT Sample Preparation Kit V2 (Illumina) following the manufacturer’s protocol. In short, genome DNA was sheared to short fragments using Covaris S220. DNA fragments were adenylate 3′ ends after end repair. Specific adapters were ligated to both ends of DNA fragments, and barcode sequence is included in one of the adaptors. Targeted size DNA fragments were selected by gel-cutting and amplified by 10-cycle PCR using universal primers (Illumina). After purification, quantification and validation, validated DNA libraries were sequenced on Illumina Sequencing System (HiSeq2000) according to the manufacturer’s paired-end (2 × 100 bp) protocol. Read pairs were aligned to the human reference genome (hg19, downloaded from the UCSC Genome Browser) by BWA version 0.7.0. Samtools version 0.1.19 was used to generate chromosomal coordinate-sorted bam files and Picard was used to remove PCR duplications. The reads were realigned around potential INDELs regions by GATK version 3.2.0 IndelRealigner following the recommended pipeline. GATK Unified Genotyper was also used to call both SNVs and INDELs. SNVs and INDELs detected by GATK were subsequently filtered by homemade pipeline, excluding: 1) all the reported mutations with low confidence; 2) germline mutations detected from blood samples; 3) mutations reported in dbSNP 138 as common SNPs (MAF > 1%).

### Transcriptome sequencing

Total RNA was extracted using the TRIzol solution (Invitrogen, Carlsbad, CA, USA), according to the manufactures’ procotols. RNA sequencing libraries were constructed using TruSeq RNA Sample Preparation Kit V2 (Illumina) following the manufacturer’s protocol. In short, RNA concentration was measured by Nanodrop and the quality was measured by agarose and Agilent 2100. RNA passed QC will begin lib prep. Purifying the poly-A containing mRNA molecules using poly-T oligo-attached magnetic beads. Following purification, the mRNA is fragmented into small pieces using divalent cations under elevated temperature. The cleaved RNA fragments are copied into first strand cDNA using reverse transcriptase and random primers. This is followed by second strand cDNA synthesis using DNA polymerase I and RNase H. These cDNA fragments then go through an end repair process, the addition of a single ‘A’ base, and then ligation of the adapters. The products are then purified and enriched with PCR (15-cycle) to create the final cDNA library. After purification, quantification and validation, validated DNA libraries were sequenced on Illumina Sequencing System (HiSeq2000) following the manufacturer’s standard workflow.

### Analysis of differentially expressed genes and fusion transcripts

The samples from non-cancerous mucosa, primary cancer and peritoneal metastasis yielded enough sequencing data of high quality by analytical QC. Transcript assembly and abundance estimation were performed to get the gene expression level. RNA-Seq reads were mapped to the human genome using TopHat (version 2.0.9, reference hg19). Cufflinks software (version 2.1.1) was used to determine the differentially expressed genes. The transcript counts for gene expression levels were calculated, and the relative transcript abundance was determined as fragments per kilo base of exon per million fragments mapped (FPKM). Raw data were extracted as FPKM values across all samples, and samples with zero values across more than 50% of the genes were excluded .Besides, detection of fusion transcripts resulting from chromosomal rearrangements was also performed for each sample. The genomic variations (SNP and INDEL) at RNA level were accessed and annotated against a collection of comprehensive functional annotation databases, including gene/protein structure, somatic variations (dbSNP, 1000 Human Genome Project, GWAS), functional consequence of amino acid change (VISIFT), known somatic mutations (COSMIC), and functional elements (transcription binding sites, conserved elements).

### Gene Ontology and Pathway Enrichment Analysis

In order to better understand the function of variant genes, we conducted a gene set enrichment analysis (GESA) for quality-controlled, filtered expression data, which conduct enrichment test genes(KEGG) biological pathways, and gene ontology (GO). Generally, a gene set with FDR < 0.1 and fold-change >1.5 was considered significantly enriched. We used gene ontology system, which covers three domains: biological processes, molecular functions and cellular components, to annotate the genes.

### RT-PCR

The selected mutated genes were further studied to validate the genomic sequencing results by quantitative RT-PCR. The genomic DNA from blood non-cancerous mucosa, primary and peritoneal metastatic tumors was used as PCR templates. PCR primers were designed for each somatic mutation using NCBI primer-blast (http://www.ncbi.nlm.nih.gov/tools/primer-blast/). Quantitative RT-PCR was performed using a routine method in our laboratory. The PCR products were sent to Sangon (Shanghai, China) for Sanger capillary sequencing. All sequencing results were aligned and visualized using chromas (http://technelysium.com.au/). All the methods were carried out in accordance with the approved guidelines.

## Additional Information

**How to cite this article**: Zhang, J. *et al.* Whole genome and transcriptome sequencing of matched primary and peritoneal metastatic gastric carcinoma. *Sci. Rep.* **5**, 13750; doi: 10.1038/srep13750 (2015).

## Supplementary Material

Supplementary Information

## Figures and Tables

**Table 1:** Fusion genes and their expression levels in triple samples.

**GeneSymbol**	**Chronic gastritis**	**Primary tumor**	**Peritoneal metastasis**	**Fold change (Primary vs gastritis)**	**Fold change (Metastasis vs gastritis)**
*GJA9-MYCBP*	23.6908	25.2078	20.8982	1.064033296	−1.133628757
*LYZ*	2108.93	1296.5	3886.12	−1.626809826	1.842697482
*DES*	84.3318	0	14.9416	/	−5.644095740
*TAGLN*	418.905	9840.69	506.742	23.49145988	1.209682386
*PCSK7*	48.5203	652.365	42.3824	13.44519716	−1.144821919
*MYH9*	84.2307	107.181	63.8822	1.272469539	−1.325918268
*C5AR2*	1.38004	0.427525	2.46365	−3.227993157	1.785201878
*MYH11*	56.3223	1557.05	11.4097	27.64535539	−4.936352400
*RHBDL2*	15.057	3.84073	9.56066	−3.920354086	−1.574891294
*OLFM4*	357.434	1376.29	0	3.850473094	/
*SMARCC1*	13.4101	11.7824	10.6925	−1.141275010	−1.574891274
*GPX4*	125.445	274.009	555.658	2.184295907	4.429494998
*FCGRT*	129.165	128.814	247.458	−1.002721929	1.915828591
*RBPMS2*	3.64694	52.0057	5.25074	14.26009202	1.439765941
*HEPHL1*	0.0199218	0.0198148	0.00958744	−1.005400014	−2.077906102
*ADAP2*	4.31958	8.13697	13.4219	1.883741012	3.107223387
*TRMT12*	4.60922	5.8219	3.88701	1.263098746	−1.185800916
*IGFBP7*	267.052	4180.75	1090	15.65519075	4.081602085
*GKN1*	0	15.4197	9.92793	/	/
*PANX1*	8.35081	6.79381	13.7156	−1.229179240	1.642427501
*OAZ2*	44.3104	71.727	55.6714	1.618739619	1.256395790
*RNF135*	8.68858	18.0227	12.2648	2.074297526	1.411600054
*MPND*	13.16	17.5939	13.1999	1.336922492	1.003031915
*CNN1*	39.8486	2542.94	16.8356	63.81503993	−2.366924857
*DHX34*	3.74313	3.63189	2.95756	−1.030628681	−1.265614223
*TFF1*	8922.76	147.509	27.6183	−60.48596847	−323.0742192
*CLDN18*	873.865	43.8734	72.5094	−19.91787740	−12.05174775
*MAP4*	23.7699	75.4865	34.2046	3.17571803	1.438987964
*RNF139*	21.9331	22.9592	38.3862	1.064033296	−1.133628757

**Table 2:** Differential expressed genes and top pathways in primary cancer and PM relative to chronic gastritis*.

**Overlap**	**Count**	**Genes**	**Top Pathways**
PrimaryDn MetastasisDn	16	*CEACAM5, PNLIPRP3, ADH1C, CYP2C19, ESRRG, HPGD, HTR1E, NKX2-2, GHRL, REG1A, CXCL5, SULT1E1, CWH43, MIXL1, NKX6-2, LIPF*	Female pregnancy, Homeobox, Triacylglycerol lipase activity
MetastasisDn MvsP.Dn	10	*FOXF1, ATP10B, GDNF, LTF, MMP7, HMP19, TMPRSS4, SLC5A7, GREM2, CCKAR*	Protease, Endopeptidase activity, Glycosylation site:N-linked (GlcNAc…)
PrimaryUp MetastasisUp	6	*LOC100505875, CDH16, NKX2-5, HOXA11, NOX4, SFRP4*	Developmental protein , Tube morphogenesis, Homeobox
MetastasisUp M vs P. up	20	*KLF14, FMO2, PCOLCE2, GHR, GPD1, KCNIP2, LIPE, LPL, NTRK2, OLR1, PCK1, PDE3B, RASD1, ITLN1, GPAM, RBP4, SAA1, SLC19A3, DGAT2,GYG2*	Triglyceride metabolic process, Neutral lipid metabolic process, Glycerolipid metabolic process

^*^Dn, Down; M, Metastasis; P, Primary.
