# Erratum: Whole genome and transcriptome sequencing of matched primary and peritoneal metastatic gastric carcinoma

**DOI:** 10.1038/srep15309

**Published:** 2015-10-20

**Authors:** J. Zhang, J. Y. Huang, Y. N. Chen, F. Yuan, H. Zhang, F. H. Yan, M. J. Wang, G. Wang, M. Su, G Lu, Y. Huang, H. Dai, J. Ji, J. Zhang, J. N. Zhang, Y. N. Jiang, S. J. Chen, Z. G. Zhu, Y. Y. Yu

Scientific Reports 5: Article number: 13750; 10.1038/srep13750 published online: 09022015; updated: 10202015.

In the original version of this Article, affiliations 1–6 were incomplete. The correct affiliations are listed below.

Affiliation 1:

Shanghai Ruijin Hospital, Shanghai Institute of Digestive Surgery and Biobank of Gastrointestinal Carcinoma, Ruijin er Road, No. 197, Shanghai, China

Affiliation 2:

Collaborative Innovation Center of Systems Biomedicine; Ruijin er Road, No. 197, Shanghai, China

Affiliation 3:

State Key Laboratory of Medical Genomics; Ruijin er Road, No. 197, Shanghai, China

Affiliation 4:

Department of Pathology of Shanghai Ruijin Hospital, Shanghai Jiao Tong University School of Medicine, Ruijin er Road, No. 197, Shanghai, China

Affiliation 5:

Department of Radiology of Shanghai Ruijin Hospital, Shanghai Jiao Tong University School of Medicine, Ruijin er Road, No. 197, Shanghai, China

Affiliation 6:

Genome Center of WuXiAppTec (Shanghai) Co., Ltd. Middle Fute Road, Shanghai

These errors have been corrected in both the HTML and PDF versions of the Article.

