# Supplementary material for: Whole genome and transcriptome sequencing of matched primary and peritoneal metastatic gastric carcinoma
Source: Sci Rep. 2015 Sep 2;5:13750. doi: 10.1038/srep13750 (PMC4557136; doi:10.1038/srep13750)
Supplement: Supplementary Information [file srep13750-s1.pdf]

Supplementary data

## Whole genome and transcriptome sequencing of matched primary and peritoneal metastatic gastric carcinoma

Zhang J[1, 2*], Huang JY[2, 3*], Chen YN[*1, 2], Yuan F[4], Zhang H[5], Yan FH[5], Wang MJ[3], Wang G[6], Su M[6], Lu G[6], Huang Y[6], Dai H[6], Ji J[1, 2], Zhang J[1, 2], Zhang JN[1, 2], Jiang YN[1, 2], Chen SJ[2, 3#], Zhu ZG[1, 2#], Yu YY[1, 2#]

Supplementary table 1. The details of WGS for blood, gastritis, primary cancer and metastatic cancer

| QC Statistics Sample | Peripheral blood | Gastritis | Primary gastric cancer | Peritoneal metastasis |
|---|---|---|---|---|
| Paired-end read length | 150*2 | 150*2 | 150*2 | 150*2 |
| Total effective data yield (Gb) | 123.473 | 101.47 | 234.93 | 211.13 |
| Total reads number (M) | 823.15 | 676.45 | 1566.2 | 1407.57 |
| Reads mapping rate | 99.81% | 99.45% | 99.67% | 99.58% |
| Properly paired mapping reads rate | 98.16% | 97.71% | 97.61% | 96.53% |
| No-mismatch mapping reads rate | 57.53% | 56.51% | 54.86% | 54.73% |
| Mismatch alignment bases rate | 0.77% | 0.73% | 0.74% | 0.81% |
| Mean coverage sequencing depth | 42 | 34 | 80 | 72 |
| Reference genome coverage | 99.09% | 99.08% | 99.09% | 99.06% |
| Reference genome coverage >= 4X | 98.70% | 98.34% | 98.79% | 98.63% |
| Reference genome coverage >= 10X | 97.84% | 96.01% | 98.17% | 97.98% |
| Reference genome coverage >= 20X | 96.02% | 85.69% | 97.85% | 97.64% |
| PCR duplication rate | 8.42% | 7.80% | 13.80% | 14.28% |

Supplementary table 2.    The somatic alterations found in chronic gastritis, primary cancer and peritoneal metastatic cancer*

| Sample | Chromosome | Position | Aelle | Alt | Depth | tumorAF | Func.refGene | Gene.refGene | ExonicFunc.refGene | cytoBand |
|---|---|---|---|---|---|---|---|---|---|---|
| Chronic gastritis | chr19 | 22155783 | C | T | 20 | 0.1 | exonic | ZNF208 | nonsynonymous SNV | 19p12 |
| Chronic gastritis | chr1 | 152382569 | A | G | 28 | 0.1429 | exonic | CRNN | nonsynonymous SNV | 1q21.3 |
| Chronic gastritis | chr14 | 92537353 | C | G | 14 | 0.8852 | exonic | ATXN3 | nonsynonymous SNV | 14q32.12 |
| Peritoneal metastatic cancer | chr2 | 74593112 | G | A | 48 | 0.08333 | exonic | DCTN1 | nonsynonymous SNV | 2p13.1 |
| Peritoneal metastatic cancer | chr8 | 10467547 | G | A | 31 | 0.09677 | exonic | RP1L1 | nonsynonymous SNV | 8p23.1 |
| Peritoneal metastatic cancer | chr12 | 11461798 | C | G | 31 | 0.1935 | exonic | PRB4 | nonsynonymous SNV | 12p13.2 |
| Peritoneal metastatic cancer | chr12 | 11506869 | C | A | 29 | 0.1034 | exonic | PRB1 | nonsynonymous SNV | 12p13.2 |
| Peritoneal metastatic cancer | chr3 | 195510252 | G | C | 17 | 0.2353 | exonic | MUC4 | nonsynonymous SNV | 3q29 |
| Peritoneal metastatic cancer | chr13 | 97484796 | G | A | 48 | 0.125 | exonic | HS6ST3 | nonsynonymous SNV | 13q32.1 |
| Peritoneal metastatic cancer | chr1 | 152382569 | A | G | 58 | 0.1552 | exonic | CRNN | nonsynonymous SNV | 1q21.3 |
| Peritoneal metastatic cancer | chr14 | 92537353 | C | G | 30 | 0.7674 | exonic | ATXN3 | nonsynonymous SNV | 14q32.12 |
| Peritoneal metastatic cancer | chr10 | 28142179 | c | T | 32 | 0.125 | exonic | ARMC4 | stopgain | 10p12.1 |
| Primary cancer | chr18 | 12325761 | G | T | 62 | 0.1129 | exonic | TUBB6 | stopgain | 18p11.21 |
| Primary cancer | chr8 | 10467547 | G | A | 27 | 0.1481 | exonic | RP1L1 | nonsynonymous SNV | 8p23.1 |
| Primary cancer | chr19 | 4511973 | A | G | 35 | 0.08571 | exonic | PLIN4 | nonsynonymous SNV | 19p13.3 |
| Primary cancer | chr1 | 155269969 | G | A | 55 | 0.1818 | exonic | PKLR | nonsynonymous SNV | 1q22 |
| Primary cancer | chr5 | 31799762 | T | C | 54 | 0.1481 | exonic | PDZD2 | nonsynonymous SNV | 5p13.3 |
| Primary cancer | chr3 | 195510251 | T | C | 13 | 0.1538 | exonic | MUC4 | nonsynonymous SNV | 3q29 |
| Primary cancer | chr7 | 100684253 | G | A | 44 | 0.09091 | exonic | MUC17 | nonsynonymous SNV | 7q22.1 |
| Primary cancer | chr21 | 27074485 | T | C | 57 | 0.1228 | exonic | JAM2 | nonsynonymous SNV | 21q21.3 |
| Primary cancer | chr16 | 31405559 | C | A | 36 | 0.1667 | exonic | ITGAD | nonsynonymous SNV | 16p11.2 |
| Primary cancer | chr15 | 78778122 | G | A | 75 | 0.1067 | exonic | IREB2 | nonsynonymous SNV | 15q25.1 |

| | | | | | | | | | | |
|---|---|---|---|---|---|---|---|---|---|---|
| Primary cancer | chr7 | 123152297 | G | A | 63 | 0.127 | exonic | IQUB | nonsynonymous SNV | 7q31.32 |
| Primary cancer | chr13 | 97484796 | G | A | 50 | 0.12 | exonic | HS6ST3 | nonsynonymous SNV | 13q32.1 |
| Primary cancer | chr10 | 124389425 | C | T | 70 | 0.1286 | exonic | DMBT1 | nonsynonymous SNV | 10q26.13 |
| Primary cancer | chr2 | 74593112 | G | A | 55 | 0.1273 | exonic | DCTN1 | nonsynonymous SNV | 2p13.1 |
| Primary cancer | chr1 | 57611003 | C | T | 60 | 0.1833 | exonic | DAB1 | nonsynonymous SNV | 1p32.2 |
| Primary cancer | chr11 | 67206144 | C | T | 36 | 0.1389 | exonic | CORO1B | nonsynonymous SNV | 11q13.2 |
| Primary cancer | chr18 | 30672840 | C | T | 33 | 0.1818 | exonic | CCDC178 | nonsynonymous SNV | 18q12.1 |
| Primary cancer | chr2 | 27850192 | C | T | 57 | 0.1579 | exonic | CCDC121 | nonsynonymous SNV | 2p23.3 |
| Primary cancer | chr14 | 92537353 | C | G | 38 | 0.839 | exonic | ATXN3 | nonsynonymous SNV | 14q32.12 |
| Primary cancer | chr9 | 112898473 | C | T | 47 | 0.1277 | exonic | AKAP2 | nonsynonymous SNV | 9q31.3 |
| Primary cancer | chr15 | 89400157 | G | C | 49 | 0.102 | exonic | ACAN | nonsynonymous SNV | 15q26.1 |
| Primary cancer | chr2 | 2.11E+08 | T | C | 59 | 0.1186 | exonic | ACADL | nonsynonymous SNV | 2q34 |

*Note:

1) All SNVs are validated by Sanger sequencing.

2) Light green background color indicates this mutation is shared in different tissue.
